# Prepubertal Exposure to Genistein Alleviates Di-(2-ethylhexyl) Phthalate Induced Testicular Oxidative Stress in Adult Rats

**DOI:** 10.1155/2014/598630

**Published:** 2014-10-29

**Authors:** Lian-Dong Zhang, He-Cheng Li, Tie Chong, Ming Gao, Jian Yin, De-Lai Fu, Qian Deng, Zi-Ming Wang

**Affiliations:** ^1^Department of Urology, The Second Affiliated Hospital, School of Medicine, Xi'an Jiaotong University, No. 157 Xiwu Road, Xi'an, Shaanxi 710004, China; ^2^Department of Nephrology, Xi'an No. 4 Hospital, Xi'an, Shaanxi 710004, China; ^3^Department of Urology, Shaanxi Provincial People's Hospital, Xi'an, Shaanxi 710068, China

## Abstract

Di-(2-ethylhexyl) phthalate (DEHP) is the most widely used plastizer in the world and can suppress testosterone production via activation of oxidative stress. Genistein (GEN) is one of the isoflavones ingredients exhibiting weak estrogenic and potentially antioxidative effects. However, study on reproductive effects following prepubertal multiple endocrine disrupters exposure has been lacking. In this study, DEHP and GEN were administrated to prepubertal male Sprague-Dawley rats by gavage from postnatal day 22 (PND22) to PND35 with vehicle control, GEN at 50 mg/kg body weight (bw)/day (G), DEHP at 50, 150, 450 mg/kg bw/day (D50, D150, D450) and their mixture (G + D50, G + D150, G + D450). On PND90, general morphometry (body weight, AGD, organ weight, and organ coefficient), testicular redox state, and testicular histology were studied. Our results indicated that DEHP could significantly decrease sex organs weight, organ coefficient, and testicular antioxidative ability, which largely depended on the dose of DEHP. However, coadministration of GEN could partially alleviate DEHP-induced reproductive injuries via enhancement of testicular antioxidative enzymes activities, which indicates that GEN has protective effects on DEHP-induced male reproductive system damage after prepubertal exposure and GEN may have promising future in its curative antioxidative role for reproductive disorders caused by other environmental endocrine disruptors.

## 1. Introduction

Endocrine disrupting chemicals (EDCs) have been proven to have potentially deleterious effects on development, growth, metabolism, and reproduction as they can interfere with the production, release, transport, metabolism, binding, action, or elimination of the natural hormones in the body [1]. In recent years, exposure to multiple EDCs simultaneously arouses great concern as the mixtures are in large amounts in the environment and how these impact on reproductive health is largely unknown. The multiple exposure effects may work in independent, dose addition, or interaction manners [2]. However, evaluation of mixture toxicity is not always simple because mechanisms of certain EDCs may be unknown and interacting effects may fluctuate quite a lot depending on dose and exposure time [3].

Prepuberty is a critical stage that is highly responsive to sex steroid actions but yet has been rarely studied, disruption due to EDCs exposure would account for a major change in the total activity of the involved hormone, resulting in adverse consequences that may be apparent during puberty as well as during adult life because of interference in the developmental programming [4, 5]. Di-2-(ethylhexyl) phthalate (DEHP) is the most widely used plasticizer in polyvinylchloride plastics, which is prevalent in cosmetics, personal care products, and medical devices, taking up about 80% of the total phthalates consumption in the world [6]. As DEHP are not chemically bound to PVC, it is easy to leach, migrate, and evaporate into indoor air and atmosphere, foodstuff, other materials, and so forth. The mechanisms by which DEHP exerts their toxic effects in male reproductive system have not been fully elucidated [7]. DEHP exerts its antiandrogen effect by suppressing foetal testosterone biosynthesis via peroxisome proliferator-activated receptors (PPARs) activation [8], in which process antioxidant enzymes were inhibited, leading to free radical production and oxidative stress which contributed to oxidative DNA damage [9]. After exposure to DEHP during prepuberty and puberty, significant decrease in GSH/GSSG redox ratio (>10-fold) and marked increase in TBARS levels were observed [10]. Epidemiological study also shows that urinary oxidative stress marker malondialdehyde (MDA) concentrations were significantly associated with several DEHP metabolite levels in prepubertal children [11].

Genistein, a weak estrogenic phytoestrogen, is widely present in the Asian diet [12] and is believed to be a potent antioxidant. It is interesting to note that genistein could enhance fertility by promoting acrosome reaction at lower doses but potentially suppresses male fertility via suppressing acrosome reaction at higher doses, having no significant effect on sperm morphology [13]. Also it is shown that isoflavones can reduce the oxidative stress induced by the other EDCs cadmium, terephthalic acid (TPA) via enhancing antioxidative defense system [14, 15], largely depending on the dose of isoflavones [16].

Although quite many studies before have examined the effects of single EDC by in vitro, ex vivo, or in vivo assays [17–19], few studies have been conducted to examine the effects of mixtures of EDCs on mammalian reproductive development and adult outcome, especially for those which act via different mechanism. So as a result of growing concerns about mixtures exposure in reality, studies on EDC mixtures exposure during prepuberty have become a focus in andrology [20].

Oxidative stress is a common pathological process involved in the mechanism of EDCs-induced testicular injury, which make it possible to have oxidative stress monitoring as an informative way to study interactions between numerous toxicants and the reproductive consequences [2, 21]. However, studies for the evaluation of the reproductive outcome and the redox state after prepubertal exposure to DEHP in combination with genistein have been lacking. In this study, we mainly investigated the changes of sexual organs weight, testis histology, testicular redox state following prepubertal exposure to mixture of DEHP, and genistein on PND90.

## 2. Materials and Methods

### 2.1. Animals and Treatment

Di-(2-ethylhexyl) phthalate (CAS: 117-81-7), was obtained from Tianjin Kermel Chemical Reagent Co., Ltd. (Tianjin, China); genistein (CAS: 446-72-0) was obtained from Shaanxi Huike Botanical Development Co., Ltd. (Xi'an, China); corn oil was obtained from Longda Co., Ltd. (Yantai, China).

Prior to study initiation, the experimental protocol was reviewed and approved by the Committee on Animal Research and Ethics of Xi'an Jiaotong University (Xi'an, China). 21-day-old specific pathogen free (SPF) Sprague-Dawley rats were obtained after weaning from the Experimental Animal Center of Xi'an Jiaotong University and were kept under 12 h light/dark cycle within the temperature range of 21 ± 2°C, relative humidity held constant at 50 ± 5%. Soy-and alfalfa-free diet (SAFD) and purified water were provided ad libitum. Every 3 rats were housed in one cage on arrival; on the next day, all the rats were treated by daily gavage from postnatal day 22 (PND22) to PND35 with corn oil (vehicle control), GEN at 50 mg/kg body weight (bw)/day (G), DEHP at 50, 150, 450 mg/kg bw/day (D50, D150, D450) and their mixture (G + D50, G + D150, G + D450); there are six rats in each group, respectively. DEHP and GEN were dissolved in corn oil and corn oil was administrated at the dose of 2 mL/kg. The dose of each chemical was calculated daily according to body weight of each rat before dosing.

The dose of genistein was chosen on the basis of previous reports [22]. Serum concentration of phytoestrogen under classical Asian diet is equivalent to that of rat at the dose of 40–50 mg/kg [23, 24] and no observed adverse effect level (NOAEL) of genistein is considered to be 50 mg/kg/day [22]. Also 50 mg/kg/day is considered to be the cutoff for low-dose effect of genistein based on the weight-of-evidence (WoE) evaluation of in vivo studies [25].

### 2.2. Body Weight, AGD, Organ Weight, and Organ Coefficient

Body weight of each rat was weighted on PND90 and anogenital distance (AGD) was measured using vernier caliper by a single investigator in a blinded manner on the same day; the AGD of each animal was divided by the cube root of body weight (AGD/body weight^1/3^) as the adjusted AGD to avoid errors caused by differences in body size. On PND90, all the rats were anaesthetized by 10% chloral hydrate. The right testis of each rat was removed and stored in −80°C refrigerator for later analysis of testicular redox state.

The left testis, seminal vesicle, prostate, and epididymis of each rat were removed and weighted separately using Mettler Toledo A2104-IC electronic balance and organ coefficient was calculated as organ weight/body weight and the left testis was immediately placed in Bouin's fixative solution for 12 h and routinely processed for histology.

### 2.3. Evaluation of Testicular Redox State

Testis tissue (200 mg) was cut into small pieces and homogenized in 1.8 mL ice-cold saline buffer (1 : 9, wt/v) using Ultra-Turrax (T8, IKA-labortechnik Staufen, Germany) to get testicular homogenates at the concentration of 0.1 g/mL. After that, testicular homogenates were centrifuged at 3,500 g for 5 min at 4°C and the supernatants were collected for further testicular redox state analysis. Total antioxidant capacity (T-AOC), superoxide dismutase (SOD) and inhibition rate, catalase (CAT), glutathione peroxidase (GSH-PX), total GSH, glutathione disulfide (GSSG), and malondialdehyde (MDA) were evaluated using the clinical chemistry assay kits (Nanjing Jiancheng Bioengineering Institute, China) according to the manufacturer's instruction to monitor testicular redox state.

T-AOC was determined by the ferric reducing/antioxidant power assay and detected at 520 nm using the spectrophotometer; the final concentration was expressed as U/mg protein.

SOD activity was measure by water soluble tetrazolium salts assay (WST-1), which monitors the inhibition rate of SOD to the process of formazan dye formation from tetrazolium salt mediated by the superoxide anion. The absorbance was scanned at 450 nm using a microplate reader.

CAT activity was determined by measuring the decomposition of H_2_O_2_ at 240 nm by ultraviolet spectrometer and the final result was expressed as U/mg protein.

GSH-PX activity was detected by determination of the reduction of GSH, the GSH reacts with 5,5-dithiobis-(2-nitrobenzoic acid) and produces yellow colored compounds which were detected at 412 nm using a spectrophotometer, and the final result was presented as U/mg protein.

T-GSH and GSSG content were measured using dithionitrobenzoic acid reagent and the absorbance was scanned at 405 nm using microplate reader, GSH content was calculated as T-GSH-2 × GSSG, and the final results were expressed as the ratio of GSH/GSSH.

MDA was analyzed using the thiobarbituric acid reactive substances (TBARS) method and the absorbance was measured with the ultraviolet spectrometer at 532 nm against blanks prepared by distilled water; result was expressed as nmol/mg protein.

### 2.4. Testicular Histology

After fixation in Bouin's fixative solution for 12 hours, testes collected on PND90 were transferred to 75% ethanol, embedded in paraffin, and cut at 5 *μ*m. Sections were stained with haematoxylin and eosin and evaluated under light microscopy. Those evaluations were performed by an experienced investigator blind to treatment.

### 2.5. Statistical Analysis

Data were expressed (mean ± SEM) and analyzed using SPSS 15.0 (SPSS Inc., Chicago, IL, USA). Normality and homogeneity of variances were evaluated prior to statistical analysis. Data were analyzed by one-way analysis of variance (ANOVA) and multiple comparison were done between combined exposure groups and control as well as single exposure groups by LSD when equal variances were assumed. When equal variances were not observed, data were analyzed by the Games-Howell Test for multiple comparisons. Differences were considered to be statistically significant at the probability level of 5% (*P* < 0.05).

## 3. Results

### 3.1. Body Weight, AGD, Organ Weight, and Organ Coefficient

**Figure 1:**
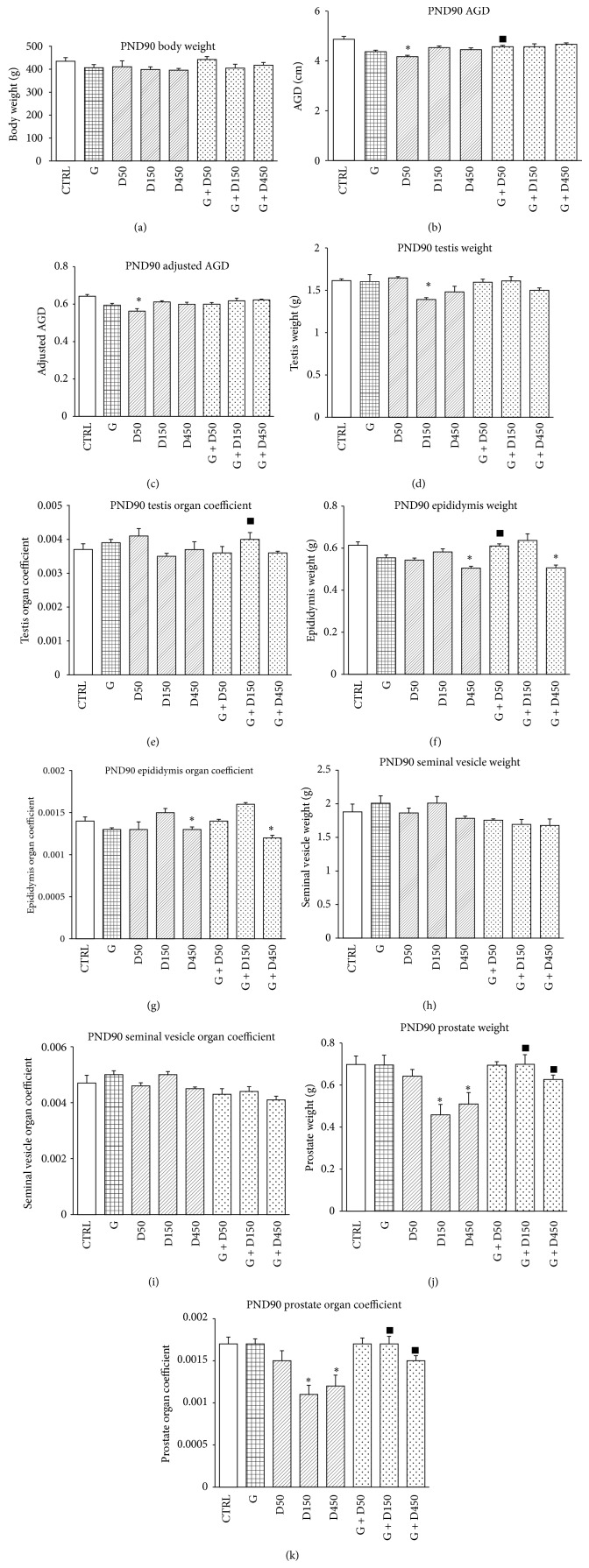
Body weight, AGD, organ weight, and organ coefficient comparison between groups on PND90. ∗: significantly different from control at *P* < 0.05; #: significantly different from G at *P* < 0.05; and ■: significantly different from corresponding D at *P* < 0.05.

Body weight, AGD, organ weight, and organ coefficient of each rat on PND90 are shown in Figure 1. No significant changes of body weight were observed in each group. Exposure to DEHP 50 mg/kg bw/day caused significant decrease in AGD and adjusted AGD compared with control (*P* < 0.05), while the combined exposure of G + D50 showed increase in AGD compared to D50 single exposure (*P* < 0.05). Rats in D150 exhibits significant difference in testis weight compared to control and significant increase was observed in group G + D150 compared to D150 single exposure (*P* < 0.05).

Both epididymis weight and epididymis organ coefficient in groups D450 and G + D450 showed significant decrease compared to control while epididymis weight of group G + D50 increased significantly compared to D50 single exposure (*P* < 0.05).

No significant differences were observed in seminal vesicle weight as well as seminal vesicle organ coefficient in all groups (*P* > 0.05). Both prostate weight and prostate organ coefficient showed decrease in groups D150 and D450 (*P* < 0.05), which were significantly lower than those in the combined exposure of G + D150 and G + D450 (*P* < 0.05).

### 3.2. Testicular Redox State

**Figure 2:**
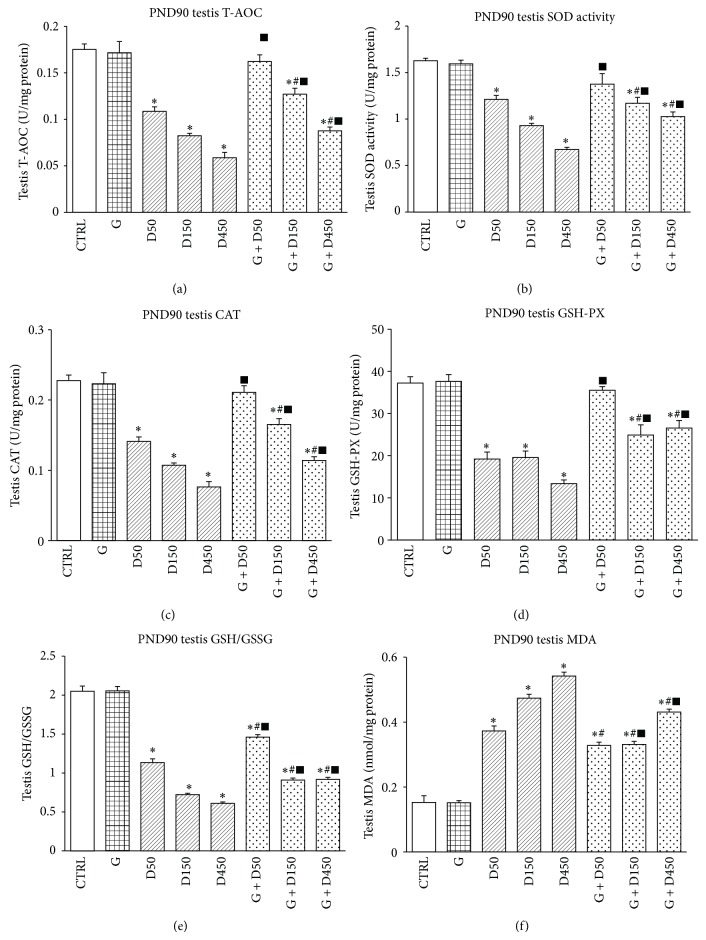
Testicular redox state comparison between groups on PND90. ∗: significantly different from control at *P* < 0.05; #: significantly different from G at *P* < 0.05; and ■: significantly different from corresponding D at *P* < 0.05.

Testicular redox state in all groups on PND90 was shown in Figure 2. Exposure to D50 and D150 as well as D450 resulted in significant reduction of testicular T-AOC, SOD activity, CAT, and GSH-PX levels as well as the ratio of GSH/GSSG (*P* < 0.05) while the combination of genistein with DEHP showed significant increase compared with corresponding single-DEHP exposure (*P* < 0.05), which indicates that genistein could partially enhance testicular antioxidative ability. In contrast, MDA level in D50, 150, and D450 increase significantly compared with control (*P* < 0.05), while the combination of genistein and DEHP showed significant decrease compared with corresponding single-DEHP exposure (*P* < 0.05), which may be largely due to the increased antioxidative capacity after genistein treatment.

### 3.3. Testicular Histology

**Figure 3:**
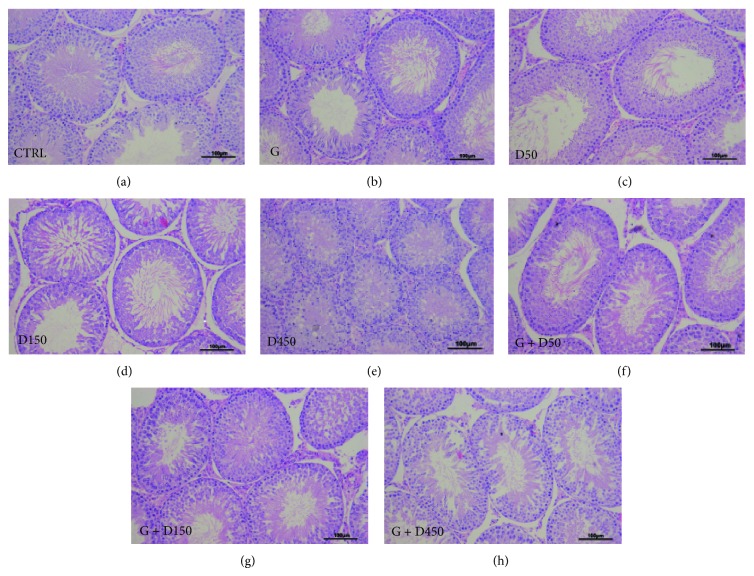
Testicular sections of male rats on PND90. Note decreased cell layers (D150), delayed formation of tubule lumen, decreased tubule diameter and the loosely arranged germ cells (D450), and improved testicular histology (G + D15 and G + D450). 200x magnification. Scale bars indicate 100 *μ*m.

Testicular sections of all groups on PND90 are shown in Figure 3. No obvious histological changes including tubule diameter, formation of tubule lumen were found after the treatment of genistein, DEHP at 50 mg/kg and their mixture and the number of cell layers in each seminiferous tubule was above 5. However, exposure to DEHP at 150 mg/kg bw/d induced the decrease of the thickness of seminiferous epithelium and the decrease of the number of cell layers, which was around 3~4 layers, while the combined exposure of G + D150 showed increased cell layer numbers compared with D150 single exposure.

Exposure to D450 exhibited delayed formation of tubule lumen and decreased tubule diameter as well as the loosely arranged germ cells, indicating that prepubertal exposure to high dose of DEHP could significantly delay tubule development of the testis during puberty and have long-term effect on adult spermatogenesis, whereas combined exposure of G + D450 showed increased cell layer number and enlargement of the tubule diameter together with formation of tubule lumen compared with D450, which indicates that prepubertal exposure to genistein could partially alleviate DEHP-induced testicular development disruption.

## 4. Discussion

Prepuberty is a critical window for male reproductive development as in this stage testicular spermatogenesis and steroidogenesis have not yet completely been established, which make male reproductive system more susceptible to EDCs [5]. The long-term effects after prepubertal EDCs exposure include compromised development of androgen-dependent sex organs due to impaired testosterone production as well as disruption of sperm motility and fertilizing ability at adulthood [10, 26]. However, there is a relative scarcity of studies focusing on prepubertal animals development and thus it is urgent to investigate the possible persistent effects on fertility and other reproductive parameters in adulthood [5]. On the other hand, the lack of studies on chemical mixtures has been an imminent concern in modern society, which make it vital to evaluate the long-term alternation of male reproductive development after prepubertal exposure.

The present study showed that combined exposure to these two common endocrine disruptors, genistein and DEHP, induced long-term alterations in testis function and development. Significant increase in AGD, testis, epididymis, and prostate weight were observed in PND90 rats treated prepubertally with both compounds compared with DEHP-single exposure. AGD or adjusted AGD is a noninvasive index of masculinization and it has been confirmed that in humans AGD is positively correlated to testis size, sperm count/fertility, penis length, and T levels, consistent with rat experimental data [27], which was closely associated with EDCs-induced male reproductive disruption during masculinization programming window [28, 29]. Here the mixture exposure of G + D450 showed no significant alternations compared with DEHP 450 while significant decrease was also observed in G + D450 compared with control, both in epididymis weight and corresponding organ coefficient, which may manifest that the combined exposure of genistein at 50 mg/kg could help the reproductive system recover, at least partially, from DEHP-induced injury, depending on the dose of DEHP during prepubertal exposure.

Oxidative stress and mitochondrial dysfunction in germ cells are suggested to attribute to phthalate-induced disruption of spermatogenesis in rodents, and Leydig cells are one of the main targets of phthalates' testicular toxicity [7]. Our results showed exposure to consecutive three doses of DEHP caused impairment of testicular antioxidative enzyme activities, alternation of the ratio of GSH/GSSG and the increase of MDA. The testicular antioxidative enzymes activities were closely related to the dose of DEHP, which showed the trend of decline as the dose increases. The most obvious histological changes, including delayed formation of tubule lumen and decreased tubule diameter as well as the loosely arranged germ cells, were observed in the D450, which showed minor deleterious damages in D150. All those outcomes indicated that prepubertal exposure to high dose of DEHP could significantly delay tubule development of the testis during puberty and have long-term effect on adult spermatogenesis. It has been confirmed that controlled and low levels of oxidative stress are essential for normal testicular function, which were generated by two vital, high energy-demanding functions, spermatogenesis and steroidogenesis. In normal physiological state, testes are equipped with potent antioxidant system that protects it against ROS damage [30]. Recent studies revealed that exposure to EDCs causes imbalance in prooxidant/antioxidant levels and thereby promotes the generation of ROS [31, 32]. DEHP has been proven to have deleterious effects on the male reproductive system via inducing dramatic changes in germ cells, Sertoli cells, and Leydig cells [33, 34]. However, the underlying mechanisms by which DEHP exerts toxic effects on reproductive system has not yet been fully elucidated. Previous studies have shown that its antiandrogenic potential is mediated by suppressing foetal testosterone biosynthesis via peroxisome proliferator-activated receptors (PPARs) activation [8], but more recently DEHP was found to induce oxidative stress in DEHP-mediated testicular dysfunction [7, 10], in which process alternations in the testicular enzymatic and nonenzymatic cellular antioxidants were involved and accompanied by elevated level of reactive oxygen species (ROS) production and DNA damage. Kasahara et al. [35] found that DEHP enhanced the generation of ROS and selectively decreased GSH and ascorbic acid in the testis, thereby inducing apoptosis of spermatocytes to cause atrophy of testis.

For combined or mixed exposures, the health effects may differ from what would be expected from simply adding or subtracting the effects of individual components, which arouse a concern that combined exposures may exhibit aberrant impact on male reproductive system, especially for those possessing “low-dose effect” [2, 25]. The combined exposure of genistein and DEHP showed upregulated antioxidative enzymes activities and downregulated MDA production compared with DEHP-single group. Even though genistein seems to help testis recover form DEHP-induced testicular injuries, the combined exposure of G + D150 and G + D450, however, still showed downregulated enzymes activities and increase of MDA level compared to control. Histological examination in mixture groups exhibited increased cell layer numbers and enlargement of the tubule diameter together with formation of tubule lumen compared with DEHP-single exposure, which indicates prepubertal exposure to genistein could partially alleviate DEHP-induced testicular development disruption. Dietary exposure of isoflavones plays an important role in various physiological processes in the body. It is well known that genistein has both estrogenic [36] and antioxidant effects [37]. Recent study revealed that genistein could improve T-AOC and catalase while decrease protein carbonyl and MDA levels in genistein-treated nephrotic rats [38]; similar findings were also reported in the other in vitro and in vivo studies [39, 40]. Although there were combined studies of genistein with other EDCs in early researches, the study on the combination of DEHP and genistein has been lacking. Gong et al. [14] revealed that genistein showed antioxidant and cytoprotective effects in the neurotoxic animal model induced by cadmium. The present data suggest that genistein could partially attenuate DEHP-induced long-term alterations in adult testis and its potency to enhance testicular antioxidative ability.

Our research demonstrated firstly that the natural phytoestrogen genistein can alleviate the deleterious effect of the most commonly used plasticizer DEHP when administrated prepubertally. Previously Yousef et al. [41] demonstrated the beneficial influences of isoflavones in reducing the negative reproductive effects of cypermethrin which were given every other day for 12 weeks in mature male New Zealand White rabbits. However, redox state evaluation was not a focus in the previous studies. In our study, for the first time, we examined redox state alternation following the three consecutive doses of DEHP and their combination with genistein. Moreover, these results suggest that prepubertal exposure to the combination of genistein and DEHP leads to long-term alterations, which were rather different from their sing effects. Thus, assessing reproductive risk based on single chemical effect might not faithfully represent the true outcome of mixture exposure during critical periods of male reproductive development. Future experiments will involve detailed analysis of cellular and molecular events contributing to long-term effects in testis development including epigenetic aberrations that may exert long-term perturbations in gene expression.

## 5. Conclusions

GEN has protective effects on DEHP-induced male reproductive system damage after prepubertal exposure and GEN may have promising future on its curative antioxidative role for reproductive disorders caused by other environmental endocrine disruptors.
